# Towards Biohydrogen Separation Using Poly(Ionic Liquid)/Ionic Liquid Composite Membranes

**DOI:** 10.3390/membranes8040124

**Published:** 2018-12-02

**Authors:** Andreia S. L. Gouveia, Lucas Ventaja, Liliana C. Tomé, Isabel M. Marrucho

**Affiliations:** 1 Centro de Química Estrutural, Instituto Superior Técnico, Universidade de Lisboa, Avenida Rovisco Pais, 1049-001 Lisboa, Portugal; andreiaslgouveia@tecnico.ulisboa.pt (A.S.L.G.); lucas.ventaja@sigma-clermont.fr (L.V.); 2 Instituto de Tecnologia Química e Biológica António Xavier, Universidade Nova de Lisboa, Av. da República, 2780-157 Oeiras, Portugal

**Keywords:** biohydrogen purification, CO_2_/H_2_ separation, PIL–IL composite membranes, gas permeation properties

## Abstract

Considering the high potential of hydrogen (H_2_) as a clean energy carrier, the implementation of high performance and cost-effective biohydrogen (bioH_2_) purification techniques is of vital importance, particularly in fuel cell applications. As membrane technology is a potentially energy-saving solution to obtain high-quality biohydrogen, the most promising poly(ionic liquid) (PIL)–ionic liquid (IL) composite membranes that had previously been studied by our group for CO_2_/N_2_ separation, containing pyrrolidinium-based PILs with fluorinated or cyano-functionalized anions, were chosen as the starting point to explore the potential of PIL–IL membranes for CO_2_/H_2_ separation. The CO_2_ and H_2_ permeation properties at the typical conditions of biohydrogen production (*T* = 308 K and 100 kPa of feed pressure) were measured and discussed. PIL–IL composites prepared with the [C(CN)_3_]^−^ anion showed higher CO_2_/H_2_ selectivity than those containing the [NTf_2_]^−^ anion. All the membranes revealed CO_2_/H_2_ separation performances above the upper bound for this specific separation, highlighting the composite incorporating 60 wt % of [C_2_mim][C(CN)_3_] IL.

## 1. Introduction

Due to its outstanding intrinsic features, hydrogen (H_2_) is considered to be the energy carrier of the future. Besides being the simplest element in the universe, the H_2_ molecule has the highest energy content per unit weight of any known fuel. However, H_2_ is not a primary fuel source, which means that it is not available in nature and thus needs to be produced [1]. Hydrogen has been produced mainly on an industrial scale from fossil fuels, through natural gas reforming or coal gasification, and from water, using electrolysis in which water (H_2_O) can be split into hydrogen and oxygen (O_2_) [2]. Although water splitting is an ecologically clean process compared to the previously mentioned H_2_ production processes, it is a highly energy-demanding technology [3].

Hydrogen production using biological processes has been attracting growing attention since it is more environmentally friendly and less energy intensive than the other described H_2_ production systems because its conditions are close to room temperature (303–313 K) and ambient pressure (100 kPa) [2]. BioH_2_ production can be divided into two main categories: light-dependent (direct or indirect biophotolysis and photo fermentation) and -independent (dark fermentation) methodologies [4,5,6]. The dark fermentation process has several advantages compared to the other biological processes namely, its potential for cost-effective hydrogen production, the high rate of cell growth, and the non-requirement of light energy [6]. In spite of the recognized potential of biohydrogen for sustainable development, there are still issues regarding its production and purification [7], such as the elimination of CO_2_, N_2_, and other impurities (H_2_O and H_2_S), so that an enriched H_2_ stream can be obtained for efficient energy generation, mostly through fuel cells [8]. Among the several methodologies for separating hydrogen, such as pressure swing adsorption (PSA), cryogenic distillation, and membrane separation, the first two are designed mainly for large-scale hydrogen production and cannot be used without modification for an upgrade of relatively small amounts of biohydrogen [9]. Thus, membrane technology has been reported as an attractive alternative for biohydrogen separation and purification [10] since it can be introduced easily into hydrogen-producing bioreactors, leading to an integrated process of bioH_2_ production and purification [11,12], not omitting its important engineering and economic advantages. Particularly, polymeric membranes, such as polysulfone (PSF) and polyimide (PI) [13], have been considered a suitable choice for biohydrogen separation as they can not only be used at the bioreactors’ operating conditions but they also have low cost, high energy efficiency, and a smaller ecological footprint than conventional separation processes [14,15,16].

Few articles have been published using membranes for bioH_2_ separation. Among them, the combination of different polymers and ionic liquids (ILs) to prepare polymer/IL composite membranes is one of the most promising approaches, which takes into account the inherent CO_2_-philicity of ILs [16]. For instance, Kanehashi et al. [17] prepared different membranes based on a fluorine-containing polyimide and different amounts of [C_4_mim][NTf_2_] IL up to 81 wt %, and studied their gas separation performance at 308 K and 100 kPa of feed pressure. The highest CO_2_/H_2_ permselectivity (6.6) was obtained for the membrane that contained the maximum amount of IL (81 wt %) [17]. Moreover, Friess et al. [18] studied the gas permeation properties at 298 K and 100 kPa of feed pressure through membranes composed of poly(vinylidene fluoride-co-hexafluoropropylene) and 20 to 80 wt % of [C_2_mim][NTf_2_] IL. Again, the largest CO_2_ permeability (533 Barrer) and CO_2_/H_2_ permselectivity (12.1) were obtained when the highest amount of IL (80 wt %) was used [18].

With the aim of designing efficient CO_2_/H_2_ separation membranes, this work explores the use of poly(ionic liquid)s (PILs), which are well recognized by their high CO_2_ affinity and designer nature [19]. Different approaches have been proposed to produce PIL-based CO_2_ separation membranes, such as neat PIL membranes [20,21,22,23], PIL copolymer membranes [19,24], and the incorporation of ILs alone or together with nanoporous materials as fillers, including zeolites or metal-organic frameworks (MOFs), to form PIL/IL/filler mixed matrix membranes (MMMs) [25,26,27]. Among all these approaches, the blend of PILs and ILs to produce homogeneous PIL–IL composite membranes is the most promising due to their high CO_2_ separation performance, as well as the good mechanical stability of the membranes [28]. Notwithstanding the potential of PILs for CO_2_ separation [28,29,30,31,32], only a reduced number of works concerning CO_2_/H_2_ separation have been reported in the literature. For instance, Carlisle et al. [33] explored the CO_2_/H_2_ separation through imidazolium PIL–IL gel membranes produced by UV polymerization. The time-lag experiments performed at room temperature and 200 kPa of feed pressure showed ideal CO_2_/H_2_ selectivities ranging from 6.6 to 12 for membranes prepared with 5 to 100 mol% of a cross-linking monomer and different amounts of free IL and IL monomer. Their best result (CO_2_ permeability of 540 Barrer and CO_2_/H_2_ selectivity of 12) was achieved for the composite containing 100 mol% of cross-linking monomer and 75 wt % of IL [33]. Moreover, Kharul et al. [34] studied the CO_2_ and H_2_ separation properties of polybenzimidazole-based PILs. The different synthesized polybenzimidazole-based PILs exhibited very similar CO_2_ and H_2_ permeabilities (<30 Barrer) and, consequently, CO_2_/H_2_ selectivities approximately equal to 1 [34].

Amongst the PIL–IL membranes developed so far for CO_2_/N_2_ separation, the most widely explored are those composed of imidazolium-based PILs with fluorinated or cyano-functionalized anions [35,36,37]. However, our group reported PIL–IL membranes made of pyrrolidinium-based PILs combining the same anions, which are particularly simple to prepare through a metathesis reaction of a commercially available polyelectrolyte. The PIL–IL membranes displayed CO_2_/N_2_ separation performances near or even above the Robeson upper bound [38,39,40,41]. In fact, the CO_2_-phylic behavior of the [NTf_2_]^−^ anion and the CO_2_ separation efficiency of the [C(CN)_3_]^−^ anion [42] motivated us to explore the most promising pyrrolidinium-based PIL–IL composites based on these two anions for CO_2_/N_2_ separation, now for CO_2_/H_2_ separation.

**Figure 1:**
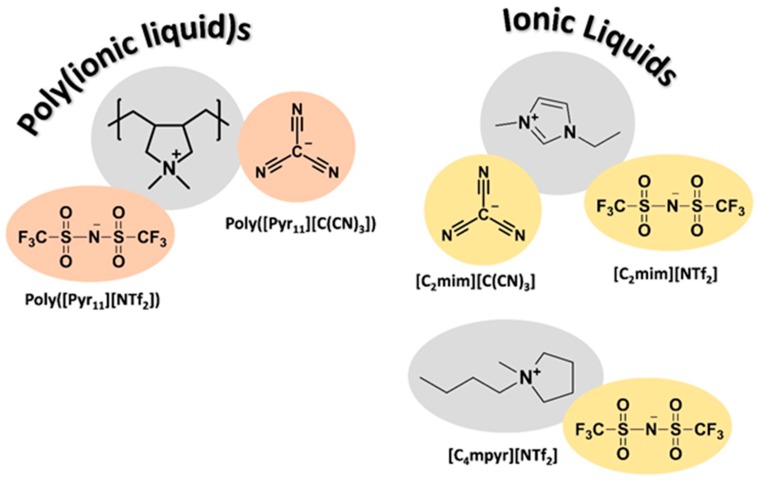
Chemical structures of the poly(ionic liquid)s (PILs) and ionic liquids (ILs) used in this work to prepare the PIL–IL membranes.

In this work, solvent casting method was used to prepare composite membranes composed of two pyrrolidinium-based PILs: poly([Pyr_11_][NTf_2_]) and poly([Pyr_11_][C(CN)_3_]). The poly([Pyr_11_][NTf_2_]) was blended with 40 and 60 wt % of the structurally similar [C_4_mpyr][NTf_2_] IL and also with 40 wt % of an imidazolium-based IL ([C_2_mim][NTf_2_]), while poly([Pyr_11_][C(CN)_3_]) was mixed with 40 and 60 wt % of [C_2_mim][C(CN)_3_] IL. The chemical structures of the PILs and ILs used are shown in Figure 1. The CO_2_ and H_2_ permeation properties (permeability, diffusivity and solubility) were determined at two different temperatures (*T* = 293 K and *T* = 308 K) under a transmembrane pressure differential of 100 kPa. A temperature of 293 K was used first so that the results obtained herein could be compared to those previously reported by our group, while *T* = 308 K was chosen to reproduce the hydrogen bioproduction conditions [13]. 

## 2. Experimental Section

### 2.1. Materials

Poly(diallyldimethylammonium) chloride solution (average *M_w_* 400,000–500,000, 20 wt % in water), acetone (99.8%), and acetonitrile (99.8%) were purchased from Sigma-Aldrich (St. Louis, MO, USA). Lithium bis(trifluoromethylsulfonyl)imide (LiNTf_2_, 99 wt % pure) and sodium tricyanomethanide (NaC(CN)_3_, 98 wt % pure) were supplied by IoLiTec GmbH (Heilbronn, Germany). The PILs used were previously synthesized by anion metathesis reactions, as described in previous studies [39,43]. All the starting materials used for PIL syntheses, as well as the organic solvents, were used as received. The water was double distilled. The ILs, 1-ethyl-3-methylimidazolium tricyanomethanide ([C_2_mim][C(CN)_3_], >98 wt % pure), 1-ethyl-3-methylimidazolium bis(trifluoromethylsulfonyl)imide ([C_2_mim][NTf_2_], >99 wt % pure), and 1-butyl-1-methylpyrrolidinium bis(trifluoromethylsulfonyl)imide ([C_4_mpyr][NTf_2_], >99 wt % pure) were provided by IoLiTec GmbH. Carbon dioxide (CO_2_) and hydrogen (H_2_) were supplied by Air Liquide with a minimum purity of 99.99%. Gases were used with no further purification.

### 2.2. Preparation of PIL–IL Membranes

Several free-standing membranes composed of the synthesized PILs and specific quantities of different ILs containing the same anions were produced by solvent casting. The first step was the preparation of 6 (*w*/*v*)% and 12 (*w*/*v*)% solutions of poly([Pyr_11_][C(CN)_3_]) and poly([Pyr_11_][NTf_2_]), respectively, in the most suitable solvents and the addition of the respective IL amounts (Table 1). The solutions were mixed using a magnetic stirrer until complete dissolution of the PIL and IL components and then poured into Petri dishes for slow evaporation of the solvent at room temperature. With the aim of obtaining homogeneous membranes, the solvent evaporation took place slowly, for 2/3 days, depending on the solvent used (Table 1), and in a saturated solvent environment. The thicknesses of the prepared membranes (70–210 μm) were measured using a digital micrometer (Mitutoyo, model MDE-25PJ, Kanagawa, Japan). Average thickness was calculated from six measurements taken at different locations of each PIL–IL membrane. All the PIL–IL membranes studied were considered to have good stability since they were malleable and flexible enough to be used in the gas permeation measurements, even for the composites with 60 wt % of IL. Moreover, the evaluation of the mechanical stability of the PIL–IL composite membranes having the [C(CN)_3_]^−^ anion was recently reported by Tomé et al. [44] (Young’s modulus (PIL–40IL) ~14 MPa; Young’s modulus (PIL–60IL) ~5).

### 2.3. Gas Permeation Experiments

A time lag equipment described in detail elsewhere [38] was used to measure and determine the ideal CO_2_ and H_2_ permeabilities and diffusivities through the prepared PIL–IL composites. Initially, each membrane was degassed under vacuum inside the permeation cell for at least 12 h before testing. The gas permeation experiments were performed at 293 K and 308 K with an upstream pressure of 100 kPa (feed) and vacuum (<0.1 kPa) as the initial downstream pressure (permeate). Three separate CO_2_ and H_2_ experiments on a single composite membrane were measured. Between each run, the permeation cell and lines were evacuated until the pressure was below 0.1 kPa.

The gas transport through the PIL–IL membranes was assumed to occur according to the solution-diffusion mass transfer mechanism [45]. Thus, the permeability (*P*) is related to diffusivity (*D*) and solubility (*S*) as follows:

(1) P=D×S

The permeate flux of each studied gas (*J_i_*) was experimentally determined using Equation (2) and assuming an ideal gas behavior and a homogeneous membrane [46]:

(2) $$J_{i}=\frac{V_{p}\Delta p_{d}}{AtRT}$$

where $V_{p}$ is the permeate volume, $\Delta p_{d}$ is the variation of downstream pressure, *A* is the effective membrane surface area, *t* is the experimental time, *R* is the gas constant, and *T* is the temperature. Equation (3) was then used to calculate the ideal gas permeability (*P_i_*) from the pressure driving force ($\Delta p_{i}$) and membrane thickness (ℓ):

(3) $$P_{i}=\frac{J_{i}}{\Delta p_{i}/\ell }$$

Gas diffusivity (*D_i_*) was determined according to Equation (4). The time-lag parameter (*θ*) was deduced by extrapolating the slope of the linear portion of the *p_d_* vs. *t* curve back to the time axis, where the intercept is equal to *θ* [47]:

(4) $$D_{i}=\frac{\ell^{2}}{6\theta }$$

After defining both *P_i_* and *D_i_*, the gas solubility (*S_i_*) was also calculated using Equation (1).

The ideal permeability selectivity (or permselectivity), $\alpha_{i/j}$, which can also be expressed as the product of the diffusivity selectivity and the solubility selectivity, was obtained by dividing the permeability of the more permeable species *i* to the permeability of the less permeable species *j*, as expressed in Equation (5):

(5) $$\alpha_{i/j}=\frac{P_{i}}{P_{j}}=\left(\frac{D_{i}}{D_{j}}\right)\times \left(\frac{S_{i}}{S_{j}}\right)$$

## 3. Results and Discussion

### 3.1. CO_2_ and H_2_ Permeation Properties

#### 3.1.1. Gas Permeability (*P*)

**Figure 2:**
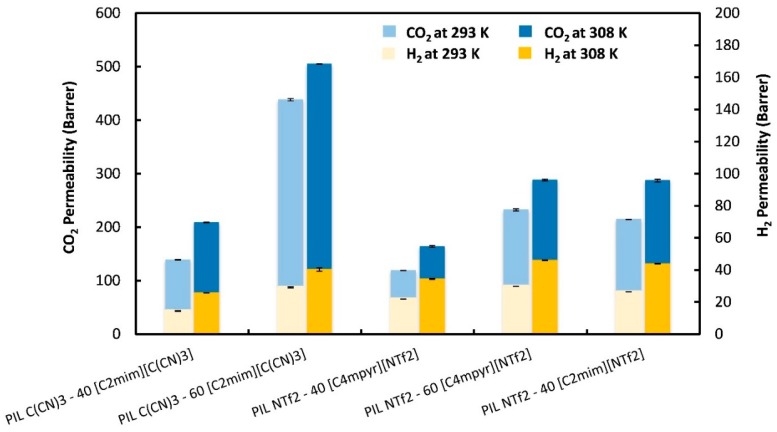
Experimental CO_2_ and H_2_ permeabilities (*P*) through the prepared PIL–IL membranes. Error bars represent standard deviations based on three experimental replicas. In some cases, the standard deviations are very small leading to error bars that cannot be clearly represented.

The CO_2_ and H_2_ permeabilities through the PIL–IL composite membranes that were studied are presented in Figure 2. The CO_2_ permeability was always higher than that of H_2_ and both permeabilities increased with increasing temperature, although the increment was not the same between the studied composites, varying from 15 to 50% for CO_2_ permeability values and from 39 to 77% for H_2_ permeabilities. The CO_2_ permeabilities at 293 K for all the membranes discussed here are in good agreement with those already reported [38,39,41], which emphasizes the high reproducibility of the method used. As expected, the incorporation of high amounts of IL led to enhanced CO_2_ and H_2_ permeabilities. Additionally, at 308 K, the temperature of bioH_2_ purification, the PIL NTf_2_–40 [C_2_mim][NTf_2_] membrane presented similar CO_2_ and H_2_ permeability values to those of PIL NTf_2_–60 [C_4_mpyr][NTf_2_]. This means that despite the higher structural compatibility of [C_4_mpyr][NTf_2_] with the pyrrolidinium-based PIL, the imidazolium-based cation of the IL is determinant in promoting increased gas permeabilities. However, and as already reported by our group [41], the use of [C_2_mim][NTf_2_] instead of [C_4_mpyr][NTf_2_] only allowed the incorporation of free IL up to 40 wt % so that a mechanically stable and homogeneous membrane could be obtained. Considering the PIL–IL membranes that comprise the [C(CN)_3_]^−^ anion in both PIL and IL, the PIL C(CN)_3_–60 [C_2_mim][C(CN)_3_] composite showed the highest CO_2_ permeability (505 Barrer) at 308 K but not the highest H_2_ permeability (40.3 Barrer), which was obtained for the PIL NTf_2_–60 [C_4_mpyr][NTf_2_] composite membrane (46.0 Barrer). Moreover, CO_2_ permeability increased about 42% while H_2_ permeability increased approximately 57% when the IL content in the PIL C(CN)_3_–[C_2_mim][C(CN)_3_] composite varied from 40 to 60 wt %. A significant difference in CO_2_ permeability (76%) was obtained for the PIL–IL membranes that contained [C_4_mpyr][NTf_2_] IL when the IL amount increased from 40 to 60 wt %, while the increment in H_2_ permeability was only around 34%.

#### 3.1.2. Gas Diffusivity (*D*)

The experimental gas diffusivity results at 293 K and 308 K through the prepared membranes are listed in Table 2. A high difference (one or, in some cases, two orders of magnitude) between CO_2_ and H_2_ diffusivity values, which corresponds to CO_2_/H_2_ diffusivity selectivities around 0.1, was observed. This difference in gas diffusivities was mainly due to the smaller size of H_2_ (2.89 Å) compared to CO_2_ kinetic diameter (3.30 Å) [48]. Moreover, both CO_2_ and H_2_ diffusivity increased with increasing temperature and with increasing IL content in the PIL–IL composite (Table 2). The same behavior was also found for CO_2_ and H_2_ permeabilities (Figure 2). From Table 2, it can also be seen that CO_2_ and H_2_ diffusivities through the prepared membranes can be ordered as follows: PIL NTf_2_–40 [C_4_mpyr][NTf_2_] < PIL NTf_2_–60 [C_4_mpyr][NTf_2_] < PIL NTf_2_–40 [C_2_mim][NTf_2_] < PIL C(CN)_3_–40 [C_2_mim][C(CN)_3_] < PIL C(CN)_3_–60 [C_2_mim][C(CN)_3_], which means that the presence of the [C(CN)_3_]^−^ anion in the composites, either in the PIL or IL’s structure, leads to higher CO_2_ and H_2_ diffusivities compared to the [NTf_2_]^−^ anion. The same trend was also observed for N_2_ diffusivities [38,39,41]. Thus, and as expected, it can be concluded that gas diffusivities follow the order of the kinetic diameters CO_2_ < N_2_ < H_2_. It can also be noted that the presence of imidazolium-based cation ([C_2_mim]^+^) in the ILs led to superior gas diffusivities compared to the pyrrolidinium-based cation ([C_4_mpyr]^+^).

Another interesting point is the comparison between gas permeability and diffusivity behaviors. Regardless of the anion, although the composite that comprised 60 wt % of IL had the highest H_2_ diffusivities (>1200 m^2^ s^−1^ at 308 K), it did not present the highest H_2_ permeabilities (Figure 2). An equivalent behavior was also obtained for the PIL NTf_2_–40 [C_4_mpyr][NTf_2_] composite membrane, which displayed the lowest H_2_ diffusivities (546 m^2^ s^−1^ at 308 K) but not the lowest H_2_ permeabilities. In the case of CO_2_, it can be seen from Table 2 and Figure 2 that CO_2_ permeability followed the same trend as CO_2_ diffusivity, with the exception of the PIL NTf_2_–60 [C_4_mpyr][NTf_2_] and PIL C(CN)_3_–40 [C_2_mim][C(CN)_3_] membranes. This behavior led us to conclude that the very high difference (three or, in some cases, four orders of magnitude) among H_2_ diffusivities is somehow compensated by a reverse behavior in H_2_ solubilities (as will be discussed in the next section), which has a significant impact on the H_2_ permeability results.

#### 3.1.3. Gas Solubility (*S*)

**Figure 3:**
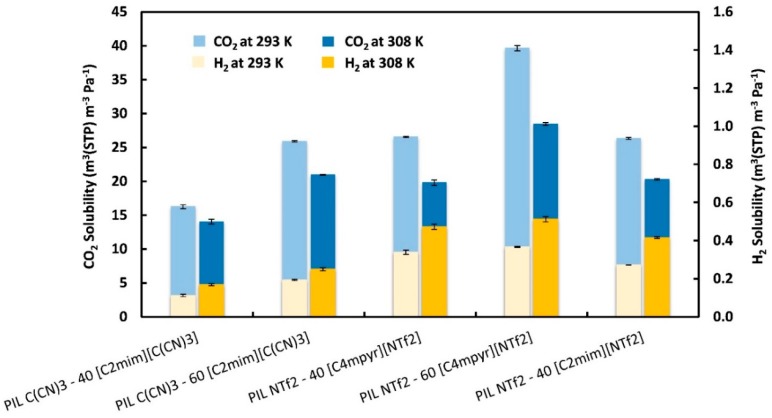
Gas solubilities (*S*) for the studied PIL–IL membranes at 293 K and 308 K.

Gas solubility (*S*) values were calculated using Equation (1) at temperatures of 293 K and 308 K and are displayed in Figure 3. The CO_2_ solubility decreased with increasing temperature while H_2_ solubility increased with increasing temperature for all the PIL–IL membranes studied. Analogous reverse H_2_ solubility behavior with temperature was also found and discussed by Raeissi et al. [49] in imidazolium-based ILs, such as [C_4_mim][NTf_2_], which means that hydrogen dissolves better at higher than at lower temperatures. This trend seems to be the general rule for H_2_ solubility in ILs [49,50,51,52,53] and has been attributed to the extreme lightness and small intermolecular forces of hydrogen molecules [49].

It can also be observed that, as expected, both CO_2_ and H_2_ solubilities were enhanced with the incorporation of high amounts of IL in the composite. For instance, when the amount of [C_2_mim][C(CN)_3_] increased from 40 to 60 wt %, the CO_2_ and H_2_ solubilities at 293 K increased almost 50% and 15%, respectively, while at 308 K the increment in CO_2_ and H_2_ solubilities was around 39% and 5%, respectively. Similar behavior was found for the PIL–IL composites comprising the [C_4_mpyr][NTf_2_] IL. Moreover, the large differences between CO_2_ and H_2_ solubilities can be explained by the high CO_2_ critical temperature (CO_2_, 31 °C; H_2_, −240 °C), corresponding to the superior condensability of CO_2_ (*T*_ε/k_ = 195.2 K) compared to H_2_ (*T*_ε/k_ = 59.7 K) [48,54]. The fact that H_2_ can almost be considered an ideal gas due to its small kinetic diameter and non-interacting nature, while CO_2_ displays a higher kinetic diameter and a quadrupole moment, also plays a role in the difference in solubilities of the two gases. For the PIL–IL composites studied in this work at 308 K, the CO_2_ solubility ranged from 14 to 28.5 (×10^−6^) m^3^_(STP)_·m^−3^·Pa^−1^ whereas the H_2_ solubility values were two orders of magnitude lower, varying from 0.17 to 0.51 (×10^−6^) m^3^_(STP)_·m^−3^·Pa^−1^. Among all the tested membranes, the PIL NTf_2_–60 [C_4_mpyr][NTf_2_] composite presented the highest CO_2_ and H_2_ solubilities at both 293 and 308 K. Regarding the influence of the anion’s structure and considering the same amount of free IL in the composite, it can be concluded that the presence of the [NTf_2_]^−^ anion in the PIL–IL membranes leads to higher CO_2_ and H_2_ solubilities compared to those membranes comprising the [C(CN)_3_]^−^ anion. As mentioned before, this behavior masks the higher H_2_ diffusivities of composites with a cyano-functionalized anion, somehow explaining the low influence of H_2_ diffusivity on the H_2_ permeability results.

### 3.2. CO_2_/H_2_ Separation Performance

The CO_2_ and H_2_ permeabilities and the ideal CO_2_/H_2_ permselectivities determined at 293 K and 308 K are summarized in Table 3. Amongst the PIL−IL membranes studied, those bearing the [C(CN)_3_]^−^ anion revealed slightly higher CO_2_/H_2_ permselectivities than those containing the [NTf_2_]^−^ anion. This behavior was also observed in our previous works concerning the use of PIL–IL membranes for CO_2_/N_2_ and CO_2_/CH_4_ separation [38,39,41]. Moreover, from Table 3, it can be seen that CO_2_/H_2_ permselectivities decreased with increasing temperature. This result can be explained by the variations in CO_2_/H_2_ solubility selectivity with temperature, which leads to a decrease in CO_2_/H_2_ permselectivity as the temperature increases [55]. In fact, CO_2_/H_2_ solubility selectivity through the studied composite membranes decreased from 78–145 at 293 K to 42–84 at 308 K. It can also be emphasized that CO_2_/H_2_ permselectivity seems to be controlled by a solubility mechanism, since CO_2_/H_2_ diffusivity selectivity (*D* CO_2_/H_2_) values were approximately equal to 0.1 at both temperatures while solubility selectivity (*S* CO_2_/H_2_) values ranged from 78–145 at 293 K and 42–84 at 308 K.

**Figure 4:**
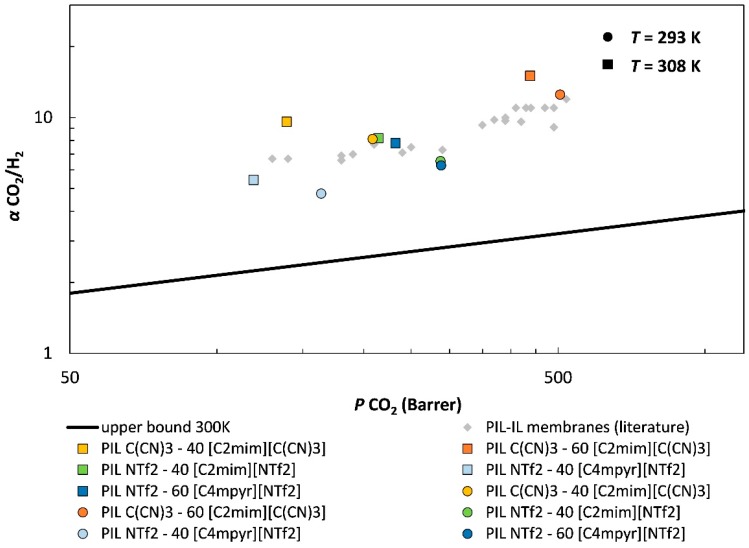
CO_2_/H_2_ separation performance of the PIL–IL membranes studied. The experimental error is within the data points. Data are plotted on a log–log scale and the upper bound at 300 K was adapted from Rowe et al. [55]. Literature data points (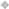) from other reported PIL–IL membranes are also displayed for comparison [33].

The gas separation performance of the studied PIL–IL membranes is shown in Figure 4, where the CO_2_/H_2_ permselectivity is plotted against the permeability of the more permeable gas species (CO_2_). This graph displays a tradeoff (black line) between gas permeability and permselectivity. These upper bound limits for several gas pairs were first developed by Robeson [56] who correlated data obtained from measurements carried out with polymeric membranes at low temperatures (298–308 K). Later, Rowe et al. [55] studied the influence of temperature on the tradeoff between gas permeability and permselectivity for different gas pairs. Thus, the upper bound at 300 K developed by Rowe et al. [55] for the CO_2_/H_2_ gas pair is represented in Figure 4 and was used to evaluate the performance of the studied PIL–IL membranes for biohydrogen purification (*T* = 308 K and 100 kPa).

Figure 4 clearly shows that all the PIL–IL membranes that were studied displayed CO_2_/H_2_ separation performances above the upper bound. The best CO_2_/H_2_ separation performance was obtained for the membrane composed of poly([Pyr_11_][C(CN)_3_]) and 60 wt % of [C_2_mim][C(CN)_3_] IL, which is in agreement with what has been observed in our recent works regarding the use of PIL–IL composites for CO_2_/N_2_ separation [39]. Literature data points for other reported PIL–IL membranes are also plotted in Figure 4 for comparison. The gas permeation measurements reported by Carlisle et al. [33] were performed at room temperature with a transmembrane pressure differential of 200 kPa. Also, their PIL–IL membranes were not prepared by the solvent casting method but through UV polymerization by mixing different percentages of imidazolium-based IL monomers, a cross-linking monomer, and free IL [33]. From Figure 4, it can be seen that the PIL–IL membranes reported in the literature also present CO_2_/H_2_ separation performances above the upper bound for the CO_2_/H_2_ gas pair at 300 K, but the PIL C(CN)_3_–60 [C_2_mim][C(CN)_3_] membrane studied in this work still revealed superior CO_2_/H_2_ separation performance.

## 4. Conclusions

In this work, dense composite membranes made of pyrrolidinium-based PILs with [C(CN)_3_]^−^ or [NTf_2_]^−^ anions and different amounts of free IL ([C_2_mim][C(CN)_3_], [C_4_mpyr][NTf_2_] or [C_2_mim][NTf_2_]) incorporated were prepared by the solvent casting method. Their CO_2_ and H_2_ permeation properties (permeability, diffusivity, and solubility) were determined at biohydrogen production conditions (*T* = 308 K and 100 kPa of feed pressure) and discussed. The CO_2_ and H_2_ permeation properties were measured at *T* = 293 K and the effect of temperature on gas separation performance was evaluated.

The PIL–IL membranes containing the [NTf_2_]^−^ anion presented the highest H_2_ permeability and solubility values, while the PIL–IL composites having the [C(CN)_3_]^−^ anion showed the highest H_2_ diffusivities and CO_2_/H_2_ permselectivities. As previously reported, increments in gas permeabilities, diffusivities, and solubilities were observed with increasing temperature and amounts of IL, with the exception of H_2_ solubility that showed the opposite behavior with temperature compared to what occurred in CO_2_ solubility. Overall, all the PIL–IL membranes studied revealed similar or superior CO_2_/H_2_ separation performance compared to the few PIL–IL composites reported so far in the literature. Particularly, at 308 K, the best result was obtained through the PIL C(CN)_3_–60 IL C(CN)_3_ composite membrane (CO_2_ permeability of 505 Barrer and CO_2_/H_2_ selectivity of 12.5), which, as shown in our previous work, also presented remarkable results for CO_2_/N_2_ separation.

## Figures and Tables

**Table 1:** Description of the poly(ionic liquid)–ionic liquid (PIL–IL) membrane’s composition and experimental preparation conditions of the solvent casting procedure.

PIL–IL Membrane	Polymer (PIL)	wt % of IL	Solvent	*T* (K)	Evaporation Time (Days)
PIL C(CN)_3_–40 [C_2_mim][C(CN)_3_]	Poly([Pyr_11_][C(CN)_3_])	40	Acetonitrile	298	3
PIL C(CN)_3_–60 [C_2_mim][C(CN)_3_]		60			
PIL NTf_2_–40 [C_4_mpyr][NTf_2_]	Poly([Pyr_11_][NTf_2_])	40	Acetone	298	2
PIL NTf_2_–60 [C_4_mpyr][NTf_2_]		60			
PIL NTf_2_–40 [C_2_mim][NTf_2_]	Poly([Pyr_11_][NTf_2_])	40	Acetone	298	2

**Table 2:** Experimental gas diffusivities (*D*) through the studied PIL–IL membranes at *T* = 293 K and *T* = 308 K.

PIL–IL Membrane	Gas Diffusivity (×10^12^) (m^2^ s^−1^) (*T* = 293 K)		Gas Diffusivity (×10^12^) (m^2^ s^−1^) (*T* = 308 K)	
	*D* CO_2_ ± σ	*D* H_2_ ± σ	*D* CO_2_ ± σ	*D* H_2_ ± σ
PIL C(CN)_3_–40 [C_2_mim][C(CN)_3_]	64 ± 1.0	970 ± 36.2	112 ± 2.5	1146 ± 34.0
PIL C(CN)_3_–60 [C_2_mim][C(CN)_3_]	127 ± 1.1	1130 ± 5.70	181 ± 0.6	1211 ± 3.2
PIL NTf_2_–40 [C_4_mpyr][NTf_2_]	34 ± 0.1	484 ± 18.5	62 ± 1.8	546 ± 20.6
PIL NTf_2_–60 [C_4_mpyr][NTf_2_]	44 ± 0.7	610 ± 6.30	76 ± 0.5	673 ± 16.9
PIL NTf_2_–40 [C_2_mim][NTf_2_]	61 ± 0.4	722 ± 1.80	106 ± 1.5	792 ± 3.70

**Table 3:** Single gas permeabilities (*P*) *^a^* and ideal permselectivities (α) of the PIL–IL membranes studied *^b^*.

PIL–IL Membrane	Gas Permeability (Barrer) (*T* = 293 K)			Gas Permeability (Barrer) (*T* = 308 K)		
	*P* CO_2_ ± σ	*P* H_2_ ± σ	α CO_2_/H_2_	*P* CO_2_ ± σ	*P* H_2_ ± σ	α CO_2_/H_2_
PIL C(CN)_3_–40 [C_2_mim][C(CN)_3_]	139 ± 0.5	14.5 ± 0.2	9.6 ± 0.2	209 ± 0.9	25.7 ± 0.1	8.1 ± 0.1
PIL C(CN)_3_–60 [C_2_mim][C(CN)_3_]	438 ± 2.1	29.1 ± 0.4	15.1 ± 0.3	505 ± 0.3	40.3 ± 1.1	12.5 ± 0.3
PIL NTf_2_–40 [C_4_mpyr][NTf_2_]	119 ± 0.2	21.9 ± 0.1	5.4 ± 0.1	164 ± 1.6	34.4 ± 0.3	4.8 ± 0.1
PIL NTf_2_–60 [C_4_mpyr][NTf_2_]	232 ± 2.2	29.8 ± 0.1	7.8 ± 0.1	288 ± 1.6	46.0 ± 0.1	6.3 ± 0.1
PIL NTf_2_–40 [C_2_mim][NTf_2_]	214 ± 0.6	26.2 ± 0.1	8.2 ± 0.1	287 ± 2.4	43.8 ± 0.2	6.5 ± 0.1

*^a^* Barrer (1 Barrer = 10^−10^ cm_(STP)_^3^·cm·cm^−2^·s^−1^·cm·Hg^−1^). *^b^* The listed uncertainties represent the standard deviations (σ) based on three experiments.

## Abbreviations

Δ*p_d_*	Variation of downstream pressure
Δ*p_i_*	Pressure driving force
*A*	Effective membrane surface area
bioH_2_	Biohydrogen
CO_2_	Carbon dioxide
*D*	Diffusivity
H_2_	Hydrogen
H_2_O	Water
H_2_S	Hydrogen sulfide
ILs	Ionic liquids
*J_i_*	Steady-state gas flux
*ℓ*	Membrane thickness
N_2_	Nitrogen
O_2_	Oxygen
*P*	Permeability
PILs	Poly(ionic liquid)s
*R*	Ideal gas law constant
*S*	Solubility
*t*	Time
*T*	Temperature
*V^p^*	Permeate volume
*α_i/j_*	Permselectivity
*θ*	Time-lag parameter
**Cations**	
[C_2_mim]^+^	1-ethyl-3-methylimidazolium
[C_4_mpyr]^+^	1-butyl-3-methylpyrrolidinium
**Anions**	
[NTf_2_]^−^	Bis(tri-fluoromethylsulfonyl)imide
[C(CN)_3_]^−^	Tricyanomethanide
